# General Patterns of Diversity in Major Marine Microeukaryote Lineages

**DOI:** 10.1371/journal.pone.0057170

**Published:** 2013-02-21

**Authors:** Massimo C. Pernice, Ramiro Logares, Laure Guillou, Ramon Massana

**Affiliations:** 1 Department of Marine Biology and Oceanography, Institut de Ciències del Mar (CSIC), Barcelona, Catalonia, Spain; 2 Station Biologique de Roscoff, Université Pierre et Marie Curie - Paris 6, Roscoff, France; 3 Laboratoire Adaptation et Diversité en Milieu Marin, CNRS, UMR 7144, Roscoff, France; J. Craig Venter Institute, United States of America

## Abstract

Microeukaryotes have vital roles for the functioning of marine ecosystems, but still some general characteristics of their current diversity and phylogeny remain unclear. Here we investigated both aspects in major oceanic microeukaryote lineages using 18S rDNA (V4–V5 hypervariable regions) sequences from public databases that derive from various marine environmental surveys. A very carefully and manually curated dataset of 8291 Sanger sequences was generated and subsequently split into 65 taxonomic groups (roughly to Class level based on KeyDNATools) prior to downstream analyses. First, we calculated genetic distances and clustered sequences into Operational Taxonomic Units (OTUs) using different distance cut-off levels. We found that most taxonomic groups had a maximum pairwise genetic distance of 0.25. Second, we used phylogenetic trees to study general evolutionary patterns. These trees confirmed our taxonomic classification and served to run Lineage Through Time (LTT) plots. LTT results indicated different cladogenesis dynamics across groups, with some displaying an early diversification and others a more recent one. Overall, our study provides an improved description of the microeukaryote diversity in the oceans in terms of genetic differentiation within groups as well as in the general phylogenetic structure. These results will be important to interpret the large amount of sequence data that is currently generated by High Throughput Sequencing technologies.

## Introduction

Decoding the complexity of marine microeukaryotic diversity is one of the biggest challenges of modern microbial ecology, given the astonishingly large diversity detected in molecular surveys [1]–[6]. Thousands of high-quality environmental Sanger sequences derived from clone libraries of the 18S rDNA genes are now available in public databases, and represent an important resource to investigate some aspects of the general architecture of protist diversity that still remain unclear. Pair-wise distances among environmental sequences are generally used to cluster them into Operational Taxonomic Units (OTUs) at different distance levels. The number of OTUs at each clustering threshold, defined here as “clustering pattern”, is a useful proxy of the diversity magnitude and it can also be used to characterize intra group distances. Clustering patterns have already been described for whole protist communities [7]–[10], but it is expected that the analysis of singular groups can highlight interesting diversity differences among lineages. These features are better reflected in the shape of phylogenetic trees from where we can infer the “phylogenetic structure” of a group, that is, the specific diversification patterns drawn by the branches (number, length and relative positions) of a phylogenetic tree [11]. Very little has been done to investigate these structures in specific groups of marine microbial eukaryotes.

The clustering pattern, based on pair-wise genetic distances, has the advantage of being easily comparable among datasets and strongly related to sequence similarity. Indeed, OTU counts provide an estimate of present diversity in each taxonomic group. Alternatively, the phylogenetic structure derived from the branching pattern of a tree gives a complementary view that contains imprints of evolutionary events occurring within given lineages. The phylogenetic structure is the result of the interplay between speciation and extinction through time, processes that are driven by factors such as geographical isolation, environmental restrictions, reproduction modes and intraspecific interactions [12]. Different protist groups may exhibit different propensities for net rate of cladogenesis (speciation minus extinction rates, [13]) over time [14], and these different evolutionary histories can influence their phylogenetic structure.

An important issue when clustering sequences in OTUs is the meaning of the clustering level applied. Several studies have attempted to identify the threshold fitting species definitions, to establish a countable unit in biodiversity inventories. Sequences sharing a similarity above 98% of the 18S rDNA gene have been proposed to derive from the same species [15], [16], but we are far from a general agreement on which value to use. Another fundamental question is identifying the maximum genetic distance that can be contained within a given phylogenetic group, regarded as a collection of species sharing the same evolutionary origin as well as several biological and ecological properties. In protist taxonomy, a relevant grouping level is the rank “Class” that targets, for instance, dinoflagellates, diatoms, and choanoflagellates. This analysis will also allow comparing traditional Classes with new ribogroups. The latter emerge from molecular surveys, do not have cultured representatives, and are dispersed throughout the eukaryotic tree of life. Significant ribogroups are the MALV within Alveolata [17], the MAST within Stramenopiles [18], and the RAD within Rhizaria [9].

Here we used publicly available 18S rDNA Sanger sequences obtained from molecular surveys aimed to study the diversity of marine planktonic protists by a culture-independent approach. We classified these sequences into separate taxonomic groups, combining classical taxonomy (Class level) with ribogrouping, and analyzed the genetic diversity in each group by OTU clustering and phylogeny. Our main objective was to get an improved representation of marine protist diversity. This will serve as a frame for interpretation and comparison with data obtained by High Throughput Sequencing (HTS) technologies like 454 or Illumina [19]. HTS sequences (that is, reads) need to be validated against data retrieved independently; otherwise they can produce strongly biased views of diversity [20], [21]. In summary, this study allowed us a) to establish the maximum genetic distance value for each taxonomic group, b) to obtain an improved picture of the diversity of different groups, and c) to get an overview of the diversification history within different lineages.

## Results

In this study we carried out an analysis of very carefully curated 18S rDNA environmental sequences derived from marine surveys both from oxic and anoxic water samples (see Table S1). A first filtering step retained 13,270 sequences of marine planktonic protists obtained from clone libraries done with universal-eukaryotic primers (Fig. S1). These were classified into 65 taxonomic groups and only sequences containing the V4–V5 regions were kept (8291 sequences; Fig. S2). Some of these groups were well-defined classical taxa (mostly at the class level) whereas the rest were ribogroups deriving exclusively from molecular environmental surveys (Table 1 and Table S2). Alveolata sequences constituted more than half of the dataset, being MALV-II (with 1815 sequences), Dinophyceae, MALV-I and Ciliophora the most represented. Stramenopiles were second in the number of sequences and included more taxonomic groups than Alveolata (21 versus 10). The largest groups within Stramenopiles were Bacillariophyceae, Chrysophyceae, MAST-3 and MAST-1. Rhizaria were represented by 682 sequences, distributed among several cercozoan and radiolarian groups. The recently proposed CCTH supergroup (Cryptophyta, Centroheliozoa, Telonemia, Haptophyta, Burki et al. [22]), was present in the dataset with 522 sequences, mainly from Prymnesiophyceae and Cryptophyceae. The remaining groups contained less than 90 sequences, with the exceptions of Choanoflagellatea and Prasinophyceae. Finally, 427 sequences remained unidentified (could not be assigned to even a supergroup), and were labeled as Novel.

**Table 1:** Classification of environmental 18S rDNA sequences in 42 taxonomic major groups.

Supergroup	Group			Distances			OTUs		
			Seq	Avg	Max	Max_c_	0.00	0.01	0.05
*Opisthokonta*	*Choanoflagellatea*	C	100	0.13	0.30	**0.24**	89	**56**	32
*Rhizaria*	*Acantharea*	C	129	0.15	0.29	**0.26**	110	**63**	29
	*Chlorarachniophyceae*	C	33	0.14	0.24	**0.23**	29	**13**	7
	*Larcopyle*	O	18	0.02	0.05	-	13	**4**	1
	*Monadofilosa*	S	81	0.11	0.30	**0.22**	72	**56**	33
	Nassellaria*	O	52	0.18	0.41	**0.32**	45	**29**	19
	*RAD A*	R	37	0.17	0.29	**0.26**	34	**23**	15
	*RAD B*	R	88	0.11	0.23	**0.16**	66	**36**	17
	*Spumellaria*	O	209	0.06	0.26	**0.13**	154	**79**	20
*Archaeplastida*	*Prasinophyceae*	C	551	0.09	0.31	**0.21**	376	**130**	30
	*Trebouxiophyceae*	C	89	0.01	0.12	**0.04**	26	**11**	6
*Stramenopiles*	*Bacillariophyceae*	C	253	0.14	0.30	**0.29**	207	**120**	57
	*Bicosoecea*	C	75	0.11	0.35	**0.28**	60	**34**	17
	*Bolidophyceae*	C	63	0.05	0.12	**0.11**	34	**12**	7
	*Chrysophyceae*	C	152	0.13	0.27	**0.24**	115	**75**	32
	*Dictyochophyceae*	C	91	0.09	0.22	**0.16**	65	**35**	16
	*Eustigmatophyceae*	C	15	0.01	0.03	-	11	**3**	1
	*Labyrinthulida*	C	29	0.17	0.35	**0.34**	26	**19**	17
	*MAST-1*	R	107	0.08	0.20	**0.16**	74	**28**	9
	*MAST-2*	R	20	0.01	0.05	-	13	**6**	2
	*MAST-3*	R	149	0.12	0.27	**0.21**	110	**73**	31
	*MAST-4*	R	92	0.03	0.07	**0.06**	60	**24**	3
	*MAST-7*	R	82	0.04	0.14	**0.08**	48	**21**	6
	*MAST-8*	R	17	0.07	0.13	-	14	**9**	6
	*MAST-12*	R	26	0.16	0.27	-	24	**19**	16
	*Oomyceta*	C	19	0.11	0.29	-	16	**13**	10
	*Pelagophyceae*	C	34	0.01	0.07	**0.02**	22	**8**	2
	*Pirsonids*	-	47	0.03	0.09	**0.08**	37	**26**	5
*CCTH*	*Cryptophyceae*	C	179	0.09	0.24	**0.21**	130	**45**	3
	*Katablepharids*	-	20	0.02	0.06	-	12	**6**	2
	*Picobiliphyceae*	R	53	0.07	0.20	**0.15**	42	**24**	8
	*Prymnesiophyceae*	C	193	0.08	0.30	**0.14**	148	**90**	37
	*Telonemia*	C	68	0.05	0.12	**0.11**	60	**42**	9
*Alveolata*	*Ciliophora*	P	956	0.18	0.42	**0.37**	788	**434**	187
	*Dinophyceae*	C	1018	0.07	0.50	**0.24**	848	**463**	122
	*MALV-I*	R	980	0.19	0.48	**0.42**	779	**431**	132
	*MALV-II*	R	1815	0.16	0.38	**0.30**	1517	**900**	353
	*MALV-III*	R	79	0.05	0.15	**0.11**	60	**38**	9
	*MALV-V*	R	51	0.02	0.07	**0.04**	41	**19**	3
*Excavata*	*Diplonemea*	C	58	0.11	0.21	**0.21**	56	**51**	27
	*Kinetoplastea*	C	40	0.23	0.39	**0.37**	31	**22**	15
*Incertae sedis*	*Apusomonadidae*	C	14	0.15	0.41	-	9	**6**	4

Each group is coded according to their taxonomic rank (S: subphylum; C: class; O: order; G: genus; R: ribogroup). The table shows the number of sequences per group (Seq), the average (Avg), maximum (Max) and maximum corrected (Max_c_) pair-wise distances, and the number of OTUs at three cut-off levels. *Nassellaria comprises also the order Collodaria.

### Justifying the target 18S rDNA region

**Figure 1:**
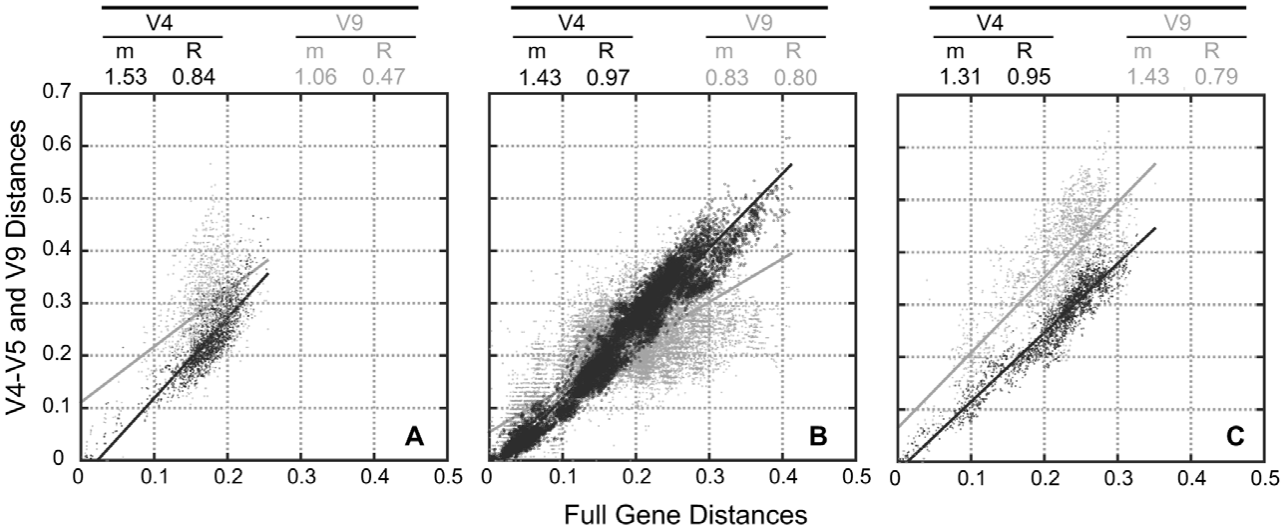
Comparison of partial and full-length 18S rDNA sequences to infer genetic distances. The three panels show pair-wise genetic distances (Jukes Cantor corrected) of the complete gene against partial regions (V4–V5 in dark grey or V9 in light grey) for sequences within Stramenopiles (A), Alveolata (B), and Rhizaria (C). Slopes (m) and coefficients (R) of the correlations are shown at the top of the graphs.

The rationale of choosing the V4–V5 region (∼550 bp) for most analyses was to maximize the number of sequences with shared positions, since many clone libraries targeted this region. We investigated how well this partial region represented the variability of the complete 18S rDNA gene. This test also included the V9 region (∼160 bp). For the three separate datasets (Stramenopiles, Alveolata and Rhizaria) we plotted the pair-wise distances calculated with the two partial regions (V4–V5 and V9) with respect to the distances computed using the full-length gene (Fig. 1). The V4–V5 region gave better results, with higher correlation coefficients (R) in the three cases (0.84 to 0.97) as compared with the values derived from the V9 region (0.47 to 0.80). In addition, the slopes of the correlation (m) were similar considering the V4–V5 region (1.31 to 1.53) whereas varied largely using the V9 region (from 0.83 to 1.43). So, this indicated that the V4–V5 region (but not the V9 region) represented well the variability of the entire 18S rDNA gene. The V4–V5 region was more variable than the complete gene, overestimating genetic distances by a factor of ∼1.4.

### Supergroup phylogenetic trees

**Figure 2:**
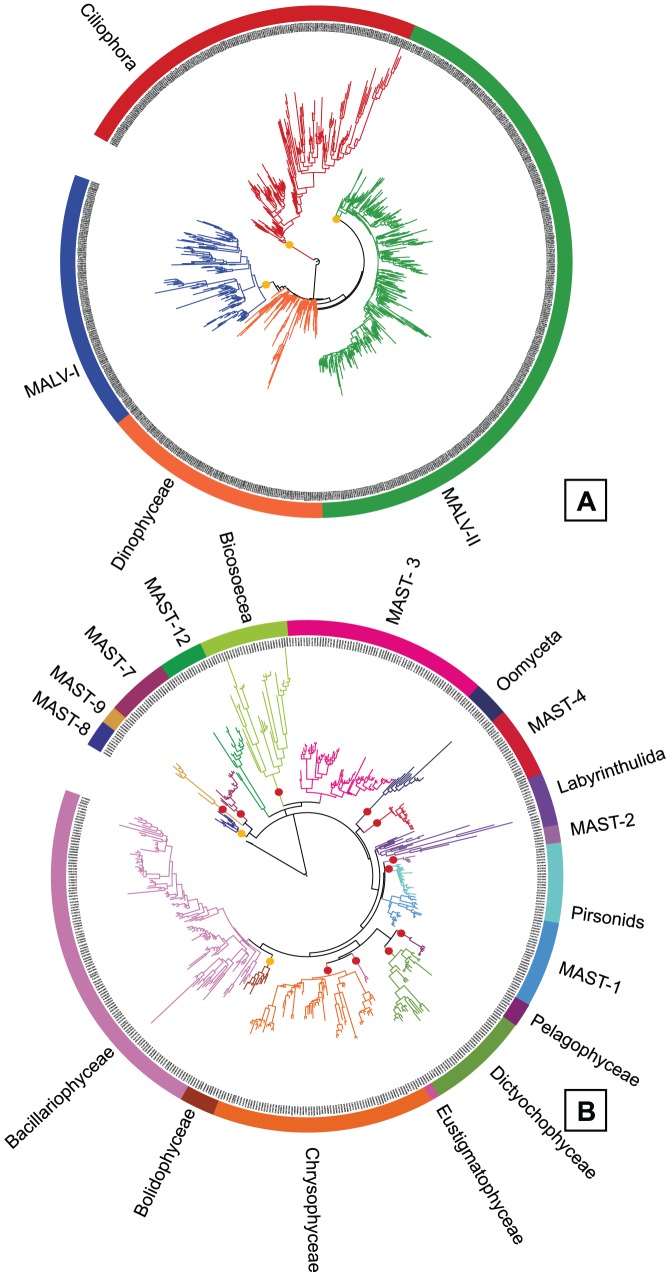
Maximum Likelihood phylogenetic trees for eukaryotic supergroups. Trees include several taxonomic groups within Alveolata (A), Stramenopiles (B), and are done with sequences representative of each OTU obtained clustering at 0.05 distance (A) and 0.01 distance (B). The number of sequences (about 550 bp in length) per tree is 798 and 523 respectively. Red dots represent bootstrap values above 75 and orange dots values above 50.

**Figure 3:**
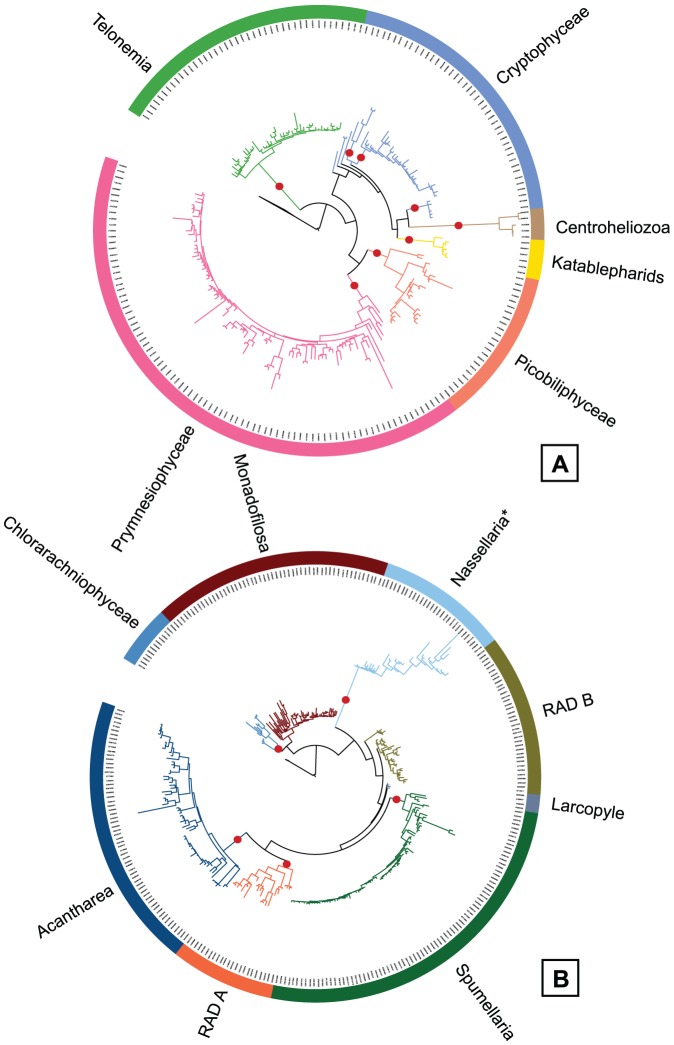
Maximum Likelihood phylogenetic trees for eukaryotic supergroups. Trees include several taxonomic groups within CCTH (A), and Rhizaria (B) and are done with sequences representative of each OTU obtained clustering at 0.05 distance. The number of sequences (about 550 bp in length) per tree 218 and 303 respectively. Red dots represent bootstrap values above 75.

Supergroup maximum-likelihood phylogenetic trees were computed to validate the taxonomic assignment of the environmental sequences. The Alveolata tree (Fig. 2A) included only the four largest groups, with one representative sequence from each OTU clustered at 0.05 distance. These groups were well recovered in the tree, but the intragroup topology was not totally correct, since MALV-I and MALV-II emerged from Dinophyceae. Probably the partial region considered (∼550 bp) was too short to resolve such a large tree. The other trees were constructed with a representative sequence of each OTU clustered at 0.01 distance. The Stramenopiles tree (Fig. 2B) displayed 18 monophyletic groups, with all photosynthetic groups (Ochrophyta) clustering together. The CCTH tree (Fig. 3A) recovered the monophyly of all groups, except Cryptophyceae. The Rhizaria tree (Fig. 3B) showed the grouping of Chlorarachniophyta and Monadofilosa (from the phylum Cercozoa), while Radiolaria was not well defined as described in previous phylogenies [23]: the class Polycystinea did not appear monophyletic and was separated into the respective orders except Collodaria and Nassellaria that were grouped (as Nassellaria*). These trees confirmed that the final dataset did not contain misclassified sequences. A nexus file of the trees is available as supporting material (Nexus file S1)

### Number of OTUs and maximum distance in taxonomic groups

The number of OTUs after clustering sequences at three different cut-off distance levels was estimated for each taxonomic group (Table 1). At 0 distance, the total number of OTUs, calculated for each group and then added up, was 6571. Using the more relaxed criterion of 0.01 distance, to take into account low-frequency sequencing errors and putative intragenomic polymorphisms, resulted in a total count of 3677 OTUs, 2301 of which belonged to Alveolata, 539 to Stramenopiles, 321 to Rhizaria and 213 to CCTH. A substantial decrease of OTUs was observed when clustering at larger distances, with a total number of 1423 OTUs at 0.05 distance.

To report the genetic distance encompassed within groups, we calculated the average, maximum, and maximum corrected pair-wise distances among all sequences within each group (Table 1). The distribution of these values, for the 20 groups having more than 29 sequences, is shown in Fig. S3. The average distance points to the typical distance between any two sequences in a group. It ranged from 0.01 (Pelagophyceae) to 0.23 (Kinetoplastea), with 75% of the cases below 0.14 (Fig. S3). The average distance is a useful descriptor, but it is the maximum distance that defines the group clustering. The intragroup maximum distance ranged from 0.07 (Pelagophyceae) to 0.50 (Dinophyceae), with 75% of the cases below 0.31. The maximum distance, however, could derive from a single highly divergent sequence, which could be fast-evolving or, more critically, could contain many sequencing errors. So we proposed another estimate, the maximum corrected distance, as the value at which 90% of sequences cluster in a single OTU. This correction was critical in groups such as Dinophyceae (decrease from 0.50 to 0.24), Prymnesiophyceae, Bolidophyceae or Prasinophyceae, whereas in others the change was minor. Seventy-five percent of the groups exhibited a maximum corrected distance below 0.25. This includes most ribogroups (all MAST clades and RAD B), indicating that these are consistent with taxonomic classes. On the other hand, the maximum corrected distance in MALV-I and MALV-II (0.42 and 0.30, respectively) suggest that these could represent higher taxonomic ranks.

### Clustering pattern of taxonomic groups

**Figure 4:**
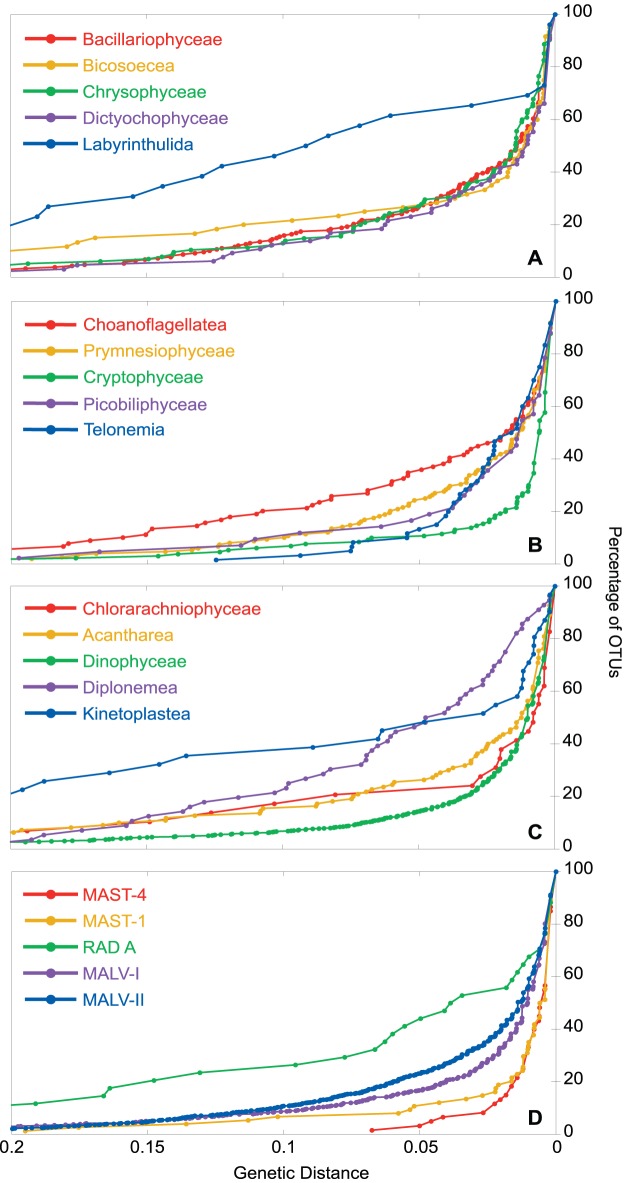
Clustering pattern of several groups of marine protists. The graphs show the percentage of OTUs when sequences are clustered at different genetic distances for several Stramenopiles groups (A), CCTH groups plus Choanoflagellatea (B), Rhizaria and Excavata groups plus Dinophyceae (C) and major ribogroups (D).

The clustering pattern was defined as the representation of the number of OTUs obtained in each group when clustering at different cut-off levels (Fig. 4). In order to compare groups, OTU counts were expressed as the percentages of the number detected at 0 distance. A high percentage of OTUs at 0.05 or 0.10 clustering distance would imply the presence of many high-rank lineages. This was the case of Labyrinthulida (Fig. 4A) that showed 65% of OTUs at a distance of 0.05. Similar examples of high-rank diversity were seen in Choanoflagellatea (Fig. 4B), Diplonemea, Kinetoplastea (Fig. 4C) and RAD A (Fig. 4D). In the opposite side of low-rank diversity were the ribogroups MAST-4 and MAST-1 (Fig. 4D), and Cryptophyceae (Fig. 4B) that yielded 2–8% OTUs at a distance of 0.05. Even containing a high number of sequences, the high-rank diversity of Dinophyceae was lower than most other groups.

### Phylogenetic structure of taxonomic groups

**Figure 5:**
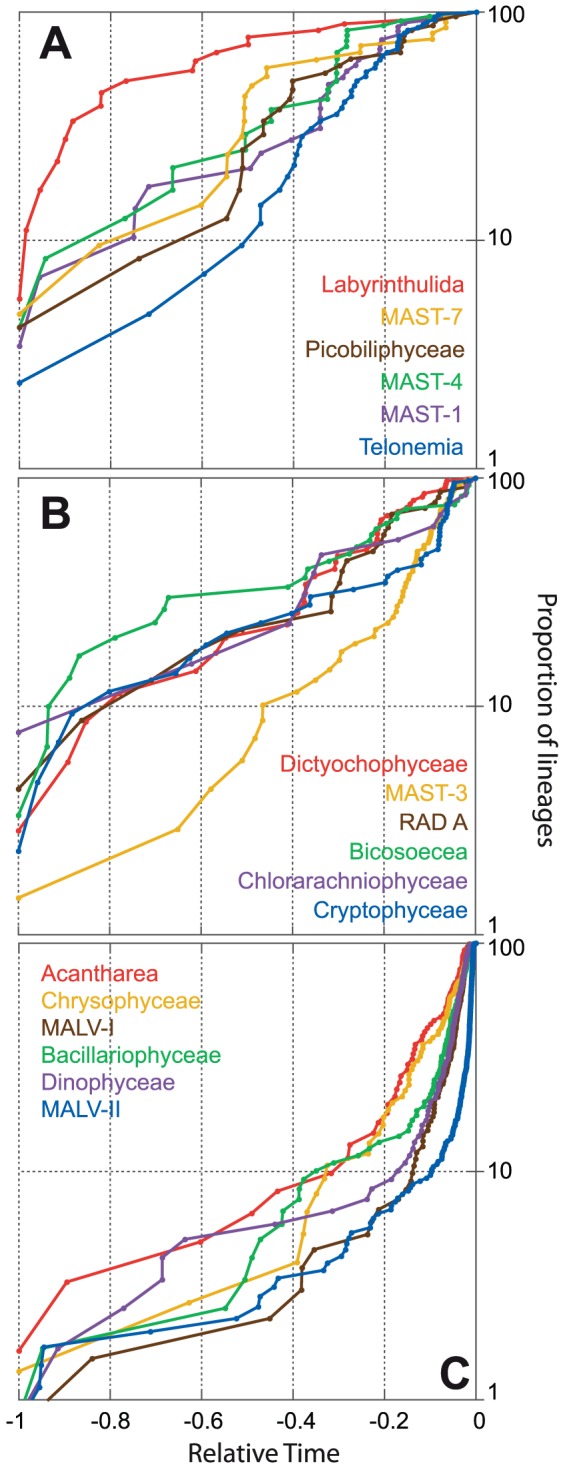
Phylogenetic structure of several groups of marine protists. Lineage Through Time (LTT) plots are based on the trees shown in Figure 2–3 and are displayed for groups having γ<0 (A), γ = 0 (B) and γ>0 (C), which indicates early, constant or late cladogenesis events, respectively. The number of lineages is standardized to the maximum number at present and relative time is considered.

Lineages Through Time (LTT) plots can be compared using the γ value, which is zero if the rate of cladogenesis was constant through time, negative if it was faster at the origin of the lineage, or positive if it was faster towards the present. Graphically, this is represented by a straight, a concave and a convex line, respectively [14]. The null hypothesis that clades diversified with a constant rate (γ = 0) was tested with one-tail test, and LLT plots were then displayed per groups that showed γ values significantly negative (Fig. 5A), positive (Fig. 5C) or non-significantly different from zero (Fig. 5B). Labyrinthulida (γ of −3.64) and MALV-II (γ of 16.72) were the two groups with most contrasting patterns, whereas RAD A and Bicosoecea were the ones closest to present a constant rate.

**Figure 6:**
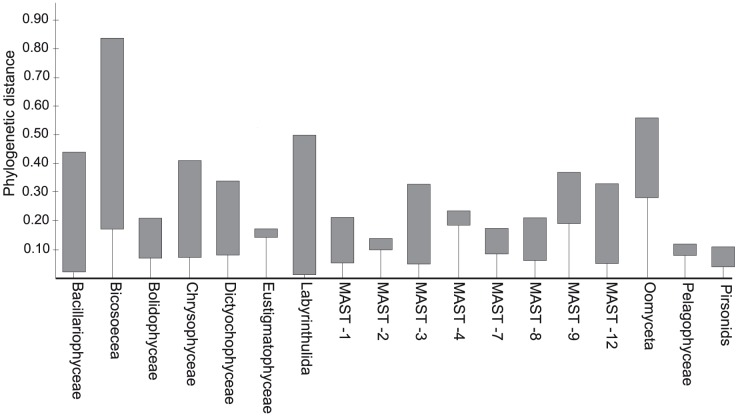
Intragroup phylogenetic distance and trunk length of Stramenopiles groups. A complementary view of phylogenetic structure of Stramenopiles is shown by displaying the trunk length (vertical lines) and the Mean Phylogenetic Distance (vertical boxes) of each group (based on tree in Figure 2B).

In order to further explore additional features contained in phylogenetic trees, we chose the Stramenopiles supergroup, since all taxonomic groups within this tree appeared monophyletic (Fig. 2B). This was done by using two descriptive parameters: the mean intragroup phylogenetic pair-wise distance (MPD) and the trunk-length (Fig. 6). There were groups characterized by large intragroup diversity and short trunks, such as Bacillariophyceae and Labyrinthulida, whereas groups like Eustigmatophyceae and MAST-4 presented the opposite structure (short diversity and long trunks). The remaining groups exhibited an intermediate position, some with very high MPD (Bicosoecea, Chrysophyceae and MAST-3) and others with low MPD (MAST-2 and Pelagophyceae). Finally, we generated a matrix of mean distances among sequences belonging to different stramenopiles (Table S3) in order to define the typical distance among groups (including both branch and trunk lengths) and to provide an idea of the phylogenetic differentiation among groups. Bicosoecea was the most isolated lineage, displaying a mean phylogenetic distance of 0.81 to the closest group. On the other hand, the parasitoid group pirsonids was the one exhibiting the lowest distance (0.24) to its closest neighbor.

## Discussion

This study is an effort to advance in the understanding of the diversity of marine protists by using publicly available 18S rDNA Sanger environmental sequences. Substantial advances have been gained by sequencing environmental genes using traditional Sanger methods, and the new High Throughput Sequencing (HTS) technologies (e.g. Illumina and 454) are now used to continue exploring marine microbial diversity [19]. Despite HTS can generate huge amounts of reads from marine microeukaryote communities, we still need a reference frame in order to interpret and organize this flood of new HTS data. Such reference frame, representing the core patterns of marine microeukaryote diversity, needs to be built based on reliable and well curated data. Despite being low-throughput, Sanger sequencing still provides probably the highest quality in sequence data. In addition, Sanger sequences are obtained in a more or less artisanal process that involves, many times, curating carefully each single sequence. For these reasons, we base our analysis in Sanger sequences only.

Our aim was to report for each taxonomic group 1) the number of OTUs and its maximum genetic distance, and 2) the evolutionary patterns inferred from phylogenetic trees. Yet, some preliminary validations were necessary before this analysis. The first step was a proper classification of environmental sequences into classical taxonomic groups or ribogroups. Phylogenetic trees indicated that chimeras or misclassified sequences, which would artificially increase intragroup diversity, were accurately removed. The second step was identifying a useful 18S rDNA region. The V4–V5 hypervariable region, widely used in environmental surveys [24], [25], provided accurate phylogenies and resulted to be a good descriptor of the variability of the entire 18S rRNA gene, overestimating pairwise distances by a factor of ∼1.4. The V9 region, optimal for early pyrosequencing technologies due to its short size [19], [26], was already known to lack specific signatures for higher-level taxa [27], and in our analysis was a poor predictor of the whole gene variability. Similar results had been obtained when comparing complete 18S rDNA and V9 regions [28] although with a lower coefficient (R^2^ = 0.40) and higher slope (m = 1.86), probably because this study did not perform a separate analysis per supergroup as we did here. The third step was to find out specific clustering cut-off levels that define taxonomic ranks. While some studies have investigated the level corresponding to the rank species [15], [16], very little has been done for higher rank categories. Regarding the clustering at the class level, 75% of the groups had a maximum corrected distance (at the V4–V5 region) below 0.25 (the full gene distance could be grossly calculated by dividing times 1.4). This was the general picture, since evolutionary rates might differ among slow- and fast-evolving lineages. Remarkably, many of the arbitrarily defined environmental ribogroups (MALV-III, MALV-V, RAD B and all MAST clades) were consistent with this maximum distance, indicating that they were congruent with a taxonomic rank equivalent to the classical class.

Once the dataset was manually curated and all sequences assigned to one of the 65 taxonomic groups, we started to analyze the diversity of the whole dataset of marine microeukaryotes. Overall, we detected 3,677 OTUs at 0.01 distance, mostly within Alveolata (63% of OTUs), Stramenopiles (15%), Rhizaria (9%) and CCTH (6%). Almost half of these OTUs belonged to taxonomically undefined ribogroups. The poor representation of the supergroups Amoebozoa and Excavata probably reflects their lower relative abundance as compared with the other supergroups in the marine plankton. This taxonomic distribution was similar to previously reviewed data [2] and could be influenced by methodological biases affecting the real proportion of taxa in natural samples. Since sequences came from libraries prepared from extracted DNA, some could derive from non-living or non-active organisms [4], [10], and taxa with high rDNA copy number could be overrepresented [29]. The moderate levels of diversity observed here were lower than what has been observed in seminal pyrosequencing studies [28], [30]. Even the groups with more sequences did not saturate, and rarefaction curves never reached a plateau (data not shown). Despite the dataset analyzed here most likely captures the general architecture of protist diversity in terms of main phylogenetic lineages, it is clear that a better estimation of diversity extent requires deeper sequencing efforts as provided by HTS. When observing how the clustering threshold affected OTU numbers, Alveolata still dominated at all levels, whereas classes like Labyrinthulida, Diplonemea and Kinetoplastea had an exceptionally high diversity. The last one exhibited the highest maximum corrected distance, probably due to a massive accumulation of sequence mutations [31].

Whereas the clustering pattern (Fig. 4) allowed quantifying the degree of genetic diversity of the groups at present time, the LTT plots (Fig. 5 and Fig. S4) used the tree topology to infer the cladogenesis events during the entire evolutionary history of different groups. It should be noted that incomplete taxon sampling could lead to the incorrect conclusion that speciation and extinction rates varied through time [32]. Other phenomena may give the false impression of non-constant rate of cladogenesis. Thus, the fact that only clades that survived to the present are considered may result in higher apparent rate of cladogenesis at the beginning of the lineage (a phenomenon known as “push of the past”), whereas higher rate of cladogenesis towards the present may be because lineages arising in recent times have had less time to go extinct (“pull of the present”) [33]. Overall, the trend of cladogenesis through time is well described by the γ value [14]. The expected tendency is to find early cladogenesis events followed by a slowdown towards the present, with γ values below 0, as commonly seen in animals and plants [34]. However, microorganisms, with their huge populations sizes (and likely lower extinction rates), may deviate from this general trend. Preliminary data showed that microbial eukaryotes had negative γ whereas prokaryotes tended to have a constant rate [14], or an increase in cladogenesis towards the present [35], although this latter trend could partly be due to the pull of the present phenomenon. Our results illustrated three evolutionary scenarios, with microeukaryote groups exhibiting early, constant, or late cladogenesis events. Thus, both Labyrinthulida and MAST-4 had early cladogenesis, even though Labyrinthulida was more diverse, perhaps because it was an early-diverging lineage [36]. Remarkably, half of the groups from our study had a positive γ (MALV-II showed the highest value), therefore deviating from the general pattern for plants and animals.

Phylogenetic supergroup trees displayed a branch distance that was not used in LTT plots, the trunk at the base of each monophyletic group. The trunk length represents the evolutionary time between the first appearance of the group and its observed diversification (putative diversifying lineages during this time are extinct). In a complete phylogeny, this trunk is a key feature to understand the intergroup diversity and complements the information given by MPD (Mean Phylogenetic Distance). Using the Stramenopiles tree as model for this analysis, it became evident that the MPD was not enough to describe the genetic isolation of a group, as confirmed by the minimum intergroup distance (Table S2). For instance, the Oomyceta had a lower MPD than Labyrinthulida and Bacillariophyceae, but a larger minimum distance (and trunk length) with its closer neighbor.

In summary, a good approximation to the evolutionary history of a given group could be reached by combining LTT plots and trunk lengths. This provided an overview of when most diversification occurred and what was the uniqueness of each group. The phylogenetic structure enriched and complemented the picture drawn by clustering pattern, which allowed reasonable comparisons among groups in terms of OTU numbers and maximum distances. Together, these two structural features gave a reasonable characterization of the diversity of the main microeukaryote clades. New sequencing technologies (pyrosequencing, Illumina) are already providing a huge amount of sequences, and a good phylogenetic and clustering pattern overview based on a robust technique is required to ensure a solid backbone for interpreting and manipulating future high-throughput datasets.

## Materials and Methods

### Sequence dataset and classification into taxonomic groups

The initial set of 163,975 sequences derived from molecular surveys of 18S rDNA genes published in GenBank until January 2010 (see Table S1) plus a few (<5%) unpublished sequences obtained at the Station Biologique de Roscoff (France). The database was filtered to keep sequences longer than 500 bp from marine planktonic protists (excluding sequences retrieved in freshwaters and sediments, or affiliating to metazoans and fungi). In addition, the sequence quality of the dataset was refined by keeping only sequences derived from clone libraries, having few unidentified bases (if any), and that passed a chimera check done with the application KeyDNATools (http://www.keydnatools.com) (Fig. S1).

The resultant 13,270 sequences were taxonomically classified with KeyDNATools (Fig. S2). Sequences ambiguously classified (less than 5 keys, keys in one region of the sequence only, or few keys from different groups [non-obvious chimeras]) were checked with BLAST [37] and assigned to a given group if they were ≥90% similar to a well-identified reference sequence. In some cases, BLAST with different parts of the sequence was done to double-check they were not chimeras. The initial dataset was distributed into 65 taxonomic groups (basically based in the “Second rank” level of Adl et al. [38]), including classical taxa mostly at the “Class” level plus new ribogroups. Sequences within each group were aligned with the FFT-NS-i strategy of MAFFT [39]. The alignment was cut manually in Seaview 3.2 [40] to keep a dataset of ∼500 bp that covered the V4–V5 regions of the 18S rDNA. Sequences shorter than 475 bp were eliminated. This process resulted in 8291 well-identified sequences plus a miscellaneous assemblage of 427 sequences that could not be placed in any taxonomic group (named Novel). A fasta file with all sequences and a text file with their affiliation are available from the authors upon request.

### Comparing different regions of the 18S rDNA

Full-length 18S rDNA sequences were prepared from three major supergroups: Rhizaria (72 sequences), Stramenopiles (60 sequences) and Alveolata (232 sequences). These were aligned with MAFFT as before and two regional alignments were extracted from the full gene alignments. The V4–V5 region was composed by the V4 region delimited by primers TAReuk454FWD1 (5′-CCAGCA(G/C)C(C/T)GCGGTAATTCC-3′, *S. cerevisiae* [U53879] positions 565–584) and TAReukREV3 (5′-ACTTTCGTTCTTGAT(C/T)(A/G)A-3′, positions 964–981) [19] and the following ∼100 bp forming the V5 region. The V9 region was delimited by primers 1391F (5′-GTACACACCGCCCGTC-3′, positions 1629–1644), and EukB (5′-TGATCCTTCTGCAGGTTCACCTAC-3′, positions 1774–1797). The V4 forward and V9 reverse primers were excluded from the alignments.

### Distance estimates and sequence clustering

Sequence alignments were processed with PAUP [41] to generate a pair-wise genetic distance matrix with Jukes-Cantor as the substitution model. The matrix was used to calculate the average distance within a group (the mean of all pair-wise distances) and also its maximum distance (the highest pair-wise distance value). The distance matrix was also used to cluster sequences in OTUs (Operational Taxonomic Units) at different distance levels with MOTHUR [42], with default settings of furthest neighbor and maximum precision (precision = 10,000). This clustering routine was also used to calculate a third estimate for each group (maximum corrected distance), which was defined as the distance at which 90% of the sequences cluster to form a single OTU.

### Phylogenetic analysis

Phylogenetic trees were constructed using one representative sequence from each OTU, generated using a clustering threshold of 0.01 (Stramenopiles, Rhizaria and CCTH) or 0.05 (Alveolata). OTU clustering was done separately for each taxonomic group, then representative sequences from the same supergroup were combined and aligned with MAFFT. Maximum-likelihood phylogenetic trees were done with RAxML [43] at the University of Oslo Bioportal (www.bioportal.uio.no), using the GTR-GAMMA evolutionary model and performing 100 alternative searches for topology and bootstrap using distinct random starting trees. Phylogenetic trees were visualized with the online tool iTOL [44]. Supergroup trees are available from the authors upon request.

For each taxonomic group within Stramenopiles, the mean phylogenetic distance (MPD) was calculated with PHYLOCOM [45]. This software was also used to estimate the length of the branch at the base of each monophyletic group, which was named “trunk”, and the average intergroup phylogenetic distance (the mean of all pair-wise distances between sequences from different groups). Phylogenetic trees representing the different taxonomic groups were extracted from the Stramenopiles tree using Dendroscope [46]. Trees were transformed to ultrametric, and used to calculate the evolution of the lineages through time (LTT). Relative time was considered, ranging from −1 (the origin of the lineage) to 0 (present time), and the number of lineages was standardized (percentage of the maximum number) to compare LTT plots among groups. For each plot, the γ-statistic was calculated as a descriptor of the evolutionary trends [32]. All analyses were carried in R environment (http://www.r-project.org/) using APE [47] and LASER [48] packages.

## Supporting Information

Figure S1 **Pipeline for database treatment.** Processing of environmental 18S rDNA sequences from initial database to working dataset, showing the number of sequences left after each filtering step.
(EPS)

Figure S2 **Pipeline for sequence treatment.** Dark grey boxes are analyses performed on the entire dataset to split sequences into 65 taxonomic groups (plus the unassigned sequences as “Novel”). Light grey boxes are analyses performed on each of the 65 groups.
(EPS)

Figure S3 **Genetic distances.** Distribution of Average, Maximum and Maximum corrected distances within the 20 classes that have more than 30 sequences.
(EPS)

Figure S4 **Phylogenetic structure of several groups of marine protists.** Lineage Through Time (LTT) plots are based on the trees shown in Figure 2–3 and are displayed for groups having γ<0 (Nassellaria-Collodaria, RAD B), γ = 0 (Bolidophyceae, Monadofilosa) and γ>0 (Spumellaria, Prymnesiophyceae, Ciliophora), which indicates early, constant or late cladogenesis events, respectively. The number of lineages is standardized to the maximum number at present and relative time is considered.
(EPS)

Table S1 **List of all studies from which we have retrieved the 18S rDNA environmental sequences.**
(DOC)

Table S2 **Classification of environmental 18S rDNA sequences in 23 taxonomic groups.** In this table are shown groups with less than 10 sequences. The groups are coded according to their taxonomic rank (D: division; P: phylum; S: subphylum; C: class; G: genus; R: ribogroup). The table shows the number of sequences per group (Seq), the average (Avg), maximum (Max) and maximum corrected (Max_c_) pair-wise distances, and the number of OTUs at three cut-off levels.
(DOC)

Table S3 **Matrix of mean distances among sequences belonging to different stramenopiles.** In bold there is the minimum distance between groups.
(DOC)

Nexus File S1 **Nexus files of the four phylogenetic trees.**
(TXT)
