# Supplementary material for: General Patterns of Diversity in Major Marine Microeukaryote Lineages
Source: PLoS One. 2013 Feb 21;8(2):e57170. doi: 10.1371/journal.pone.0057170 (PMC3578791; doi:10.1371/journal.pone.0057170)
Supplement: Table S1 — List of all studies from which we have retrieved the 18S rDNA environmental sequences. (DOC) [file pone.0057170.s005.doc]

| **Year** | **First author** | **Reference** | **Size fraction (µm)** |
| --- | --- | --- | --- |
| 2001 | Díez | Appl Environ Microbiol **68**:4554-4558 | 0.2 - 2 |
| 2001 | López-García | Nature **409**:603-607 | 0.2 - 5 |
| 2001 | Moon-van der Staay | Nature **409**:607-610 | 0.2 - 3 |
| 2003 | Stoeck | Appl Environ Microbiol **69**:5656-5663 | Whole |
| 2004 | Corredor | Appl Environ Microbiol **70**:5459-5468 | Whole |
| 2004 | Massana | Appl Environ Microbiol **70**:3528-3534 | 0.2 -3 |
| 2004 | Romari | Limnol Oceanogr **49**: 784-798 | 0.2 - 3 |
| 2004 | Savin | Microb Ecol **48**: 51-65 | 5 - 100 |
| 2004 | Yuan | FEMS Microbiol Let **240**: 163-170 | Whole |
| 2006 | Behnke | Appl Environ Microbiol **72**:3626-3636 | Whole |
| 2006 | Lovejoy | Appl Environ Microbiol **72**:3085-3095 | 0.2 - 3 |
| 2006 | Massana | Aquat Microb Ecol **45**:171-180 | 0.2 - 3 |
| 2006 | Medlin | Microb Ecol **52**: 53-71 | 0.2 - 3 |
| 2006 | Stoeck | Protist **157**: 31-43 | Whole |
| 2006 | Worden | Aquat Microb Ecol **43**:165-175 | 0.45 - 2 |
| 2006 | Zuendorf | FEMS Microbiol Ecol **58**: 476-491 | Whole |
| 2007 | Bass | Proc Roy Soc Lond B **274**: 3069-3077 | Whole |
| 2007 | Countway | Environ Microbiol **9**: 1219-1232 | 0.2 - 200 |
| 2007 | López-García | Environ Microbiol **9**: 546-554 | Whole |
| 2007 | Massana | Environ Microbiol **9**: 2260-2269 | 0.2 - 3 |
| 2007 | Not | Environ Microbiol **9**: 1233-1252 | 0.2 - 2 |
| 2007 | Stoeck | Microb Ecol **53**: 328-339 | Whole |
| 2008 | Amaral-Zettler | Environ Sci Technol **42**: 9072-9080 | Whole |
| 2008 | Guillou | Environ Microbiol **10**:3349-3365 | Various |
| 2008 | Not | Deep Sea Res Part I **55**: 1456-1473 | 0.2 - 3 |
| 2009 | Alexander | Environ Microbiol **11**: 360-381 | Whole |
| 2009 | Amacher | Deep Sea Res Part I **56**: 2206-2215 | Whole |
| 2009 | Caron | Appl Environ Microbiol **75**:5797-5808 | 0.2 - 200 |
| 2009 | Luo | Hydrobiologia **636**: 233-248 | 0.2 – 50 / Whole |
| 2009 | Not | PLoS ONE **4**:e7143 | 0.6 - 3 |
| 2009 | Potvin | J Eukaryot Microbiol **56**: 174-181 | 0.2 - 3 |
| 2009 | Shi | PLoS ONE **4**:e7657 | 0.2 - 3 |
| 2009 | Terrado | Aquat Microb Ecol **56**:25-39 | 0.2 – 3 / 3 - Whole |
| 2010 | del Campo | Prot.**162**: 435-448 | 0.2 - 3 |

**Table S1.** **List of all studies from which we have retrieved the 18S rDNA environmental sequences.**
