# Supplementary material for: General Patterns of Diversity in Major Marine Microeukaryote Lineages
Source: PLoS One. 2013 Feb 21;8(2):e57170. doi: 10.1371/journal.pone.0057170 (PMC3578791; doi:10.1371/journal.pone.0057170)
Supplement: Table S2 — Classification of environmental 18S rDNA sequences in 23 taxonomic groups. In this table are shown groups with less than 10 sequences. The groups are coded according to their taxonomic rank (D: division; P: phylum; S: subphylum; C: class; G: genus; R: ribogroup). The table shows the number of sequences per group (Seq), the average (Avg), maximum (Max) and maximum corrected (Maxc) pair-wise distances, and the number of OTUs at three cut-off levels. (DOC) [file pone.0057170.s006.doc]

| **Supergroup** | **Group** |  |  | **Distances** | | | **OTUs** | | |
| --- | --- | --- | --- | --- | --- | --- | --- | --- | --- |
|  |  |  | Seq | Avg | Max | Max_c_ | 100% | 99% | 95% |
| *Amoebozoa* | *Breviata* | G | 3 | 0.19 | 0.27 | - | 3 | **3** | 3 |
|  | *Lobosa* | D | 8 | 0.31 | 0.52 | - | 8 | **7** | 5 |
|  | *Other Amoebozoa* | - | 1 | - | - | - | 1 | **1** | 1 |
|  | *Ichthyosporea* | C | 1 | - | - | - | 1 | **1** | 1 |
| *Rhizaria* | *Endomyxa* | S | 3 | 0.27 | 0.30 | - | 3 | **3** | 3 |
|  | *Other Cercozoa* | - | 31 | - | - | - | 23 | **15** | 12 |
| *Archaeplastida* | *Chlorophyceae* | C | 5 | 0.08 | 0.13 | - | 5 | **5** | 4 |
|  | *Embryophyceae* | C | 6 | 0.13 | 0.26 | - | 6 | **3** | 3 |
|  | *Florideophyceae* | C | 1 | - | - | - | 1 | **1** | 1 |
|  | *Ulvophyceae* | C | 1 | - | - | - | 1 | **1** | 1 |
| *Stramenopiles* | *MAST-9* | R | 8 | 0.08 | 0.17 | - | 8 | **6** | 4 |
|  | *Phaeophyceae* | C | 3 | 0.02 | 0.03 | - | 3 | **3** | 1 |
|  | *Planomonadida* | C | 1 | - | - | - | 1 | **1** | 1 |
|  | *Raphidophyceae* | C | 2 | - | 0.01 | - | 2 | **1** | 1 |
|  | *Xanthophyceae* | C | 1 | - | - | - | 1 | **1** | 1 |
| *CCTH* | *Centroheliozoa* | C | 8 | 0.06 | 0.11 | - | 7 | **5** | 2 |
|  | *Pavlovophyceae* | C | 1 | - | - | - | 1 | **1** | 1 |
| *Alveolata* | *Apicomplexa* | C | 6 | 0.28 | 0.42 | - | 6 | **6** | 6 |
|  | *Ellobiopsidae* | C | 1 | - | - | - | 1 | **1** | 1 |
|  | *MALV-IV* | R | 5 | 0.05 | 0.09 | - | 5 | **5** | 3 |
|  | *Perkinsea* | C | 4 | 0.13 | 0.16 | - | 4 | **4** | 4 |
| *Excavata* | *Eopharyngia* | C | 3 | 0.00 | 0.00 | - | 3 | **1** | 1 |
|  | *Jacobea* | C | 6 | 0.20 | 0.44 | - | 6 | **4** | 3 |

**Table S2. Classification of environmental 18S rDNA sequences in 23 taxonomic groups**. In this table are shown groups with less than 10 sequences. The groups are coded according to their taxonomic rank (D: division; P: phylum; S: subphylum; C: class; G: genus; R: ribogroup). The table shows the number of sequences per group (Seq), the average (Avg), maximum (Max) and maximum corrected (Max_c_) pair-wise distances, and the number of OTUs at three cut-off levels.
