# Supplementary material for: General Patterns of Diversity in Major Marine Microeukaryote Lineages
Source: PLoS One. 2013 Feb 21;8(2):e57170. doi: 10.1371/journal.pone.0057170 (PMC3578791; doi:10.1371/journal.pone.0057170)
Supplement: Table S3 — Matrix of mean distances among sequences belonging to different stramenopiles. In bold there is the minimum distance between groups. (DOC) [file pone.0057170.s007.doc]

|  | Bicosoecida | Bolidophyceae | Chrysophyceae | Dictyophyceae | Eustigmatophyceae | Labyrinthulidae | MAST1 | MAST2 | MAST3 | MAST4 | MAST7 | MAST8 | MAST9 | MAST12 | Oomyceta | Pelagophyceae | Pirsonia |
| --- | --- | --- | --- | --- | --- | --- | --- | --- | --- | --- | --- | --- | --- | --- | --- | --- | --- |
| Bacillariophyta | 1.05 | 0.47 | 0.63 | 0.7 | 0.52 | 0.65 | 0.49 | 0.48 | 0.68 | 0.65 | 0.69 | 0.6 | 0.92 | 0.85 | 0.83 | 0.6 | **0.45** |
| Bicosoecida | 0 | 0.94 | 1.04 | 1.11 | 0.93 | 0.97 | 0.86 | 0.85 | **0.81** | 0.91 | 0.92 | 0.84 | 1.16 | 1.02 | 1.08 | 1.01 | 0.82 |
| Bolidophyceae | 0.94 | 0 | 0.52 | 0.59 | 0.41 | 0.53 | 0.38 | 0.36 | 0.57 | 0.54 | 0.57 | 0.48 | 0.81 | 0.74 | 0.71 | 0.49 | **0.34** |
| Chrysophyceae | 1.04 | 0.52 | 0 | 0.62 | **0.42** | 0.63 | 0.48 | 0.47 | 0.67 | 0.64 | 0.68 | 0.59 | 0.91 | 0.84 | 0.81 | 0.52 | 0.44 |
| Dictyophyceae | 1.11 | 0.59 | 0.62 | 0 | 0.52 | 0.71 | 0.56 | 0.54 | 0.75 | 0.71 | 0.75 | 0.66 | 0.98 | 0.91 | 0.89 | **0.42** | 0.51 |
| Eustigmatophyceae | 0.93 | 0.41 | 0.42 | 0.52 | 0 | 0.53 | 0.38 | 0.36 | 0.57 | 0.53 | 0.57 | 0.48 | 0.8 | 0.73 | 0.71 | 0.42 | **0.33** |
| Labyrinthulidae | 0.97 | 0.53 | 0.63 | 0.71 | 0.53 | 0 | 0.46 | 0.44 | 0.61 | 0.57 | 0.61 | 0.52 | 0.84 | 0.78 | 0.75 | 0.61 | **0.42** |
| MAST1 | 0.86 | 0.38 | 0.48 | 0.56 | 0.38 | 0.46 | 0 | 0.28 | 0.5 | 0.47 | 0.5 | 0.41 | 0.73 | 0.67 | 0.64 | 0.46 | **0.25** |
| MAST2 | 0.85 | 0.36 | 0.47 | 0.54 | 0.36 | 0.44 | 0.28 | 0 | 0.48 | 0.45 | 0.49 | 0.4 | 0.72 | 0.65 | 0.62 | 0.44 | **0.24** |
| MAST3 | 0.81 | 0.57 | 0.67 | 0.75 | 0.57 | 0.61 | 0.5 | 0.48 | 0 | 0.54 | 0.56 | 0.47 | 0.79 | 0.65 | 0.72 | 0.65 | **0.46** |
| MAST4 | 0.91 | 0.54 | 0.64 | 0.71 | 0.53 | 0.57 | 0.47 | 0.45 | 0.54 | 0 | 0.55 | 0.46 | 0.78 | 0.71 | 0.62 | 0.61 | **0.42** |
| MAST7 | 0.92 | 0.57 | 0.68 | 0.75 | 0.57 | 0.61 | 0.5 | 0.49 | 0.56 | 0.55 | 0 | **0.38** | 0.61 | 0.73 | 0.72 | 0.65 | 0.46 |
| MAST8 | 0.84 | 0.48 | 0.59 | 0.66 | 0.48 | 0.52 | 0.41 | 0.4 | 0.47 | 0.46 | 0.38 | 0 | 0.62 | 0.64 | 0.63 | 0.56 | **0.37** |
| MAST9 | 1.16 | 0.81 | 0.91 | 0.98 | 0.8 | 0.84 | 0.73 | 0.72 | 0.79 | 0.78 | **0.61** | 0.62 | 0 | 0.96 | 0.95 | 0.88 | 0.69 |
| MAST12 | 1.02 | 0.74 | 0.84 | 0.91 | 0.73 | 0.78 | 0.67 | 0.65 | 0.65 | 0.71 | 0.73 | 0.64 | 0.96 | 0 | 0.88 | 0.81 | **0.62** |
| Oomyceta | 1.08 | 0.71 | 0.81 | 0.89 | 0.71 | 0.75 | 0.64 | 0.62 | 0.72 | 0.62 | 0.72 | 0.63 | 0.95 | 0.88 | 0 | 0.79 | **0.6** |
| Pelagophyceae | 1.01 | 0.49 | 0.52 | 0.42 | 0.42 | 0.61 | 0.46 | 0.44 | 0.65 | 0.61 | 0.65 | 0.56 | 0.88 | 0.81 | 0.79 | 0 | **0.41** |
| Pirsonia | 0.82 | 0.34 | 0.44 | 0.51 | 0.33 | 0.42 | 0.25 | **0.24** | 0.46 | 0.42 | 0.46 | 0.37 | 0.69 | 0.62 | 0.6 | 0.41 | 0 |

**Table S3. Matrix of mean distances among sequences belonging to different stramenopiles.** In bold there is the minimum distance between groups
